# Gene correlation network analysis to identify regulatory factors in sepsis

**DOI:** 10.1186/s12967-020-02561-z

**Published:** 2020-10-08

**Authors:** Zhongheng Zhang, Lin Chen, Ping Xu, Lifeng Xing, Yucai Hong, Pengpeng Chen

**Affiliations:** 1 Department of Emergency Medicine, Sir Run Run Shaw Hospital, Zhejiang University School of Medicine, No 3, East Qingchun Road, Hangzhou, 310016 Zhejiang Province China; 2 Department of Critical Care Medicine, Affiliated Jinhua Hospital, Zhejiang University School of Medicine, Jinhua, China; 3 Emergency Department, Zigong Fourth People’s Hospital, 19 Tanmulin Road, Zigong, Sichuan China

**Keywords:** Sepsis; intensive care unit, Gene co-expression netwoek, Mortality

## Abstract

**Background and objectives:**

Sepsis is a leading cause of mortality and morbidity in the intensive care unit. Regulatory mechanisms underlying the disease progression and prognosis are largely unknown. The study aimed to identify master regulators of mortality-related modules, providing potential therapeutic target for further translational experiments.

**Methods:**

The dataset GSE65682 from the Gene Expression Omnibus (GEO) database was utilized for bioinformatic analysis. Consensus weighted gene co-expression netwoek analysis (WGCNA) was performed to identify modules of sepsis. The module most significantly associated with mortality were further analyzed for the identification of master regulators of transcription factors and miRNA.

**Results:**

A total number of 682 subjects with various causes of sepsis were included for consensus WGCNA analysis, which identified 27 modules. The network was well preserved among different causes of sepsis. Two modules designated as black and light yellow module were found to be associated with mortality outcome. Key regulators of the black and light yellow modules were the transcription factor CEBPB (normalized enrichment score = 5.53) and ETV6 (NES = 6), respectively. The top 5 miRNA regulated the most number of genes were hsa-miR-335-5p (n = 59), hsa-miR-26b-5p (n = 57), hsa-miR-16-5p (n = 44), hsa-miR-17-5p (n = 42), and hsa-miR-124-3p (n = 38). Clustering analysis in 2-dimension space derived from manifold learning identified two subclasses of sepsis, which showed significant association with survival in Cox proportional hazard model (*p* = 0.018).

**Conclusions:**

The present study showed that the black and light-yellow modules were significantly associated with mortality outcome. Master regulators of the module included transcription factor CEBPB and ETV6. miRNA-target interactions identified significantly enriched miRNA.

## Background

Sepsis is defined as organ dysfunction syndrome caused by uncontrolled inflammatory response to infection. Sepsis is a leading cause of mortality in hospitalized patients [1, 2], and accounts for 30% of case fatality in hospitalized patients [3]. Despite the high mortality and morbidity, few agents are proven to be effective for the treatment of sepsis. Thus, more regulatory factors need to be identified to provide potential targets for the design of effective therapeutic agents.

Several studies have used transcriptome analysis to investigate potential biological pathways regulating the pathogenesis of sepsis [4–8]. These studies were performed by differential gene expression analysis, followed by enrichment analyses to established functional pathways. In these analyses, genes were tested individually. The sensitivity to identify biologically meaningful genes can be low due to multiple testing adjustment. Sepsis is a heterogeneous syndrome and its pathogenesis involves hundreds of genes. In this situation, the individual contribution of a single gene is too small to be detected with univariate test. In most diseases, genes function via networks of co-expressed genes with similar biological functions. Thus, identification of co-expression pattern could provide further insights into sepsis-associated biological pathways. Weighted gene co-expression network analysis (WGCNA) is a systems biology approach used for finding gene clusters with highly correlated expression levels and for relating them to phenotypic traits [9]. Rather than relating thousands of genes to the clinical trait, WGCNA focuses on the relationship between a few modules and the trait [10, 11]. To the best of our knowledge, WGCNA has been used to explore coexpression pattern in mouse model of sepsis [12], HIV infection [13], in vitro inflammatory cells [14], and pediatric sepsis [15]. Regulatory factors including transcription factors and miRNA were not systematically explored in adult sepsis.

The present study aimed to identify gene co-expression modules in sepsis by using the consensus WGCNA (consensus from different causes of sepsis including pneumonia and abdominal sepsis). These consensus modules were related to clinical traits and enriched to functional biological pathways. Potential regulators of these modules were explored by using well curated databases.

## Materials and methods

### The GEO dataset and data preprocessing

The study used the publicly avaiable dataset GSE65682 from the Gene Expression Omnibus (GEO) database. The dataset contained 802 samples including healthy controls, non-sepsis critically ill patients and sepsis patients. Futhermore, the sepsis patients could be further categorized into pneumonia sepsis (n = 192), abdominal sepsis (n = 51) and others (n = 443) based on infection site. PAXgene blood RNA was isolated at intensive-care unit (ICU) admission and whole-blood leukocyte transcriptome was performed at the platform of Affymetrix Human Genome U219 Array. Key benefits of the gene chip include increased productivity and efficiency through parallel processing, excellent gene expression accuracy and reproducibility and complete coverage of the annotated genome. Further detials of the dataset can be found at other publications [16, 17]. Raw intensity expression data were preprocessed with the Robust Multi-array Average (RMA) method [18]. An advantage of this method is that normalization occurs at the probe level (rather than at the probeset level) across all of the selected hybridizations. The maximum expression intensity was used when multiple probe sets mapped an individual gene symbol. The quality of processed data were checked by using MA plot (Additional file 1: Figure S1).

### Consensus weighted gene co-expression network analysis

The first step in constructing a consensus WGCNA was to choose the soft threshoulding power to which co-expression similarity was raised to calculte adjaency. We chose from a set of values from 4 to 20 based on the criterion of approximate scale-free topology [10]. Since the topological overlap matrices (TOM) of different sepsis may have different statistical property, we performed quantile normalization over the three types of sepsis (e.g. pneumonia, abdomianl and other sepsis). The consensus TOM was calculated by taking the component-wise (“parallel”) minimum of the TOMs in individual dataset, which was then input to hierarchical clustering. Finally, modules were identified in the resulting dendrogram using the Dynamic Tree Cut algorithm [19]. This algorithm has several advantages such as capability of identifying nested clusters and flexibility. Modules with similar expression profiles were merged at the threshold of 0.25.

Gene significance was defined as the Student t-test statistic for testing differential expression between sepsis and healthy controls. The significance level was adjusted for multiple testing with Bonferroni correction. The dataset also contained critically ill patients such as those with major abdominal surgery without infection. There were common pathways between critical illness and severe infection, the comparison between sepsis versus non-infectious critical illness would omit some important genes. Thus, the differential expression was tested between sepsis versus healthy controls.

### Relating consensus module to pneumonia-specific sepsis module

Modules specific to pnenomia sepsis were identified by the method as described above. Pneumonia sepsis modules were then related to consensus modules. We calculated the overlaps of each pair of pneumonia-consensus modules, and used the hypergeometric test to assign a p-value to each of the pairwise overlaps. This is also known as the cross-tabulation based comparison of modules. This method is justified by the idea that if a module is well preserved and reproducible in all types of sepsis (pneumonia, abdominal and other sepsis), this module could represent the common pathways involving the pathogenesis of sepsis [20].

Module preservation across all three datasets were explored by pairwise comparing eigengene networks in penumonia, abdominal and other sepsis. Mortality was added as an additional “eigengene”. Network preservation is simply the difference between adjacencies in the two compared sets [20]. A small difference of the adjacency matrix between two sets indicate the modules are well preserved between the two comparing sets.

### Relating consensus modules to clinical traits

Module eigengene was calculated for each module as the first principal component of gene expressions for that module. Correlation analysis was performed to relate module eigengene to external traits including age, gender, mortality and survival time (e.g. survivors were censored at 28 days). We combined three datasets into one and performed correlation analysis.

Gene significance for mortality was correlated to the module membership to investigate whether genes significantly associated with mortality outcome was also associated with module membership. Module membership (eigengene-based connectivity) for each gene was calculated by correlating its gene expression profile with the module eigengene of a given module. For a given module, a module membership value of 0 indicates that a gene is not part of the module; whereas a module memberhsip of − 1 or 1 is highly connected to the module.

### Enrichment analysis for biological function and transcription factors

Modules associated with important clinical trait such as mortality were further analyzed for their enrichment in Gene Ontology (GO) pathways [21]. Specifically, the gene set from a given module were enriched to GO terms to find whether some of functional GO terms are over-represented using annotations for that gene set. Upset plot was employed to display overlapped genes among different GO terms. Dotplot shows the gene ratio and adjusted p values for each enriched GO terms. Enriched terms were organized into a network with edges connecting overlapping gene sets. In this way, mutually overlapping gene sets are tend to cluster together, making it easy to identify functional modules. The category netplot depicts the linkages of genes and GO terms as a network, which is helpful to see which genes are involved in enriched pathways and genes that may belong to multiple annotation categories.

Modules (gene lists) significantly correlated with the mortality trait were tested for its over-representation in transcription factor (TF) binding motifs by using RcisTarget [22]. Two types of databases (i.e. Gene-motif rankings and the annotation of motifs to transcription factors) were employed in the analysis: Gene-motif rankings which provides the rankings of all the genes for each motif and the annotation of motifs to transcription factors. Parameter settings for the score of each pair of gene-motif were: species = Homo sapiens, Scoring/search space = 500 bp uptream the transcription start site (TSS), Number of orthologous species = 10. The annotation of motifs to transcription factors was performed using the motifAnnotations_hgnc ('mc9nr', 24,453 motifs).

### Identification of miRNA-target interactions

The multiMiR package was employed for the retrieval of miRNA-target interactions from 14 external databases in R. These databases are comprehensive collections of predicted and validated miRNA-target interactions and their associations with diseases and drugs [23]. The module of interest was those associated with mortlaity outcome. It was interesting to check whether some, or all, of these genes within a module were targeted by the same miRNA(s). We restrited our search to the “mirtarbase” table because this table included only experimentally validated miRNA-target interactions.

### Survival analysis

The association of each module with the survival outcome was determined by using Cox proportional model. The eigengene value of each module was added into the Cox regression model for univariate analysis. Genes matched to the module with the strongest association with survival were used to cluster patients into two groups using reversed graph embedding (DDRTree), which projects data into a reduced dimensional space while constructs a principal tree which passes through the middle of the data simultaneously [24]. Samples were clustered into two groups using k-means clustering. Survival probablity of the two groups were comapred using the log-rank test.

## Results

### Demographic data

A total of 802 subjects were initially identified from the GEO database. 116 subjects were excluded because they were either healthy or non-infectious controls, and 4 subjects were excluded because they were outliers (Additional file 1: Figure S2). As a result, a number of 682 subjects were included in our analysis. These subjects were classified by the infection site into pneumonia sepsis, abdominal sepsis and unselected sepsis (other sepsis). Demographic data were comparable between the three groups (Table 1). The median age was 63 years (IQR: 53 to 72), and 58% (394/682) patients were male. The overall 28 day mortality rate was 17% (113/682).

**Table 1:** Demographic data on the study population stratified by the causes of sepsis

Variables	Total (n = 682)	Abdominal sepsis (n = 48)	Other sepsis (n = 442)	Pneumonia sepsis (n = 192)	*p*
Age, median (IQR)	63 (53, 72)	64 (54, 68.75)	63 (53, 73)	63 (53, 72)	0.977
Sex, n (%)					0.538
Female	288 (42)	23 (48)	189 (43)	76 (40)	
Male	394 (58)	25 (52)	253 (57)	116 (60)	
Follow up days, Median (IQR)	28 (28, 28)	28 (28, 28)	28 (20.5, 28)	28 (28, 28)	0.176
Mortality, n (%)	113 (17)	7 (15)	66 (15)	40 (21)	0.172

*IQR* interquartile range;

### Consensus Network construction and module detection

**Fig. 1:**
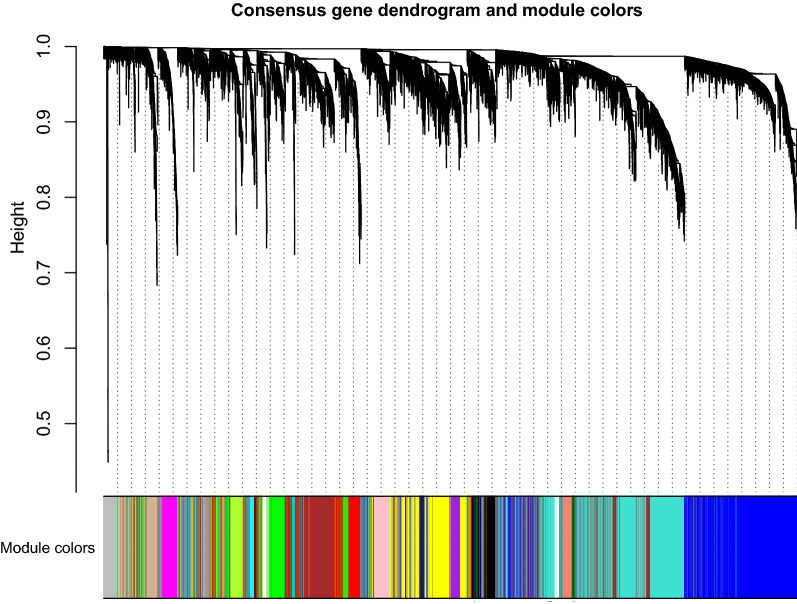
Consensus weighted gene coexporession network analysis. Modules were identified in the resulting dendrogram using the Dynamic Tree Cut algorithm. A total of 27 modules were identified. Modules were distinguished from each other by assigning different colors

**Fig. 2:**
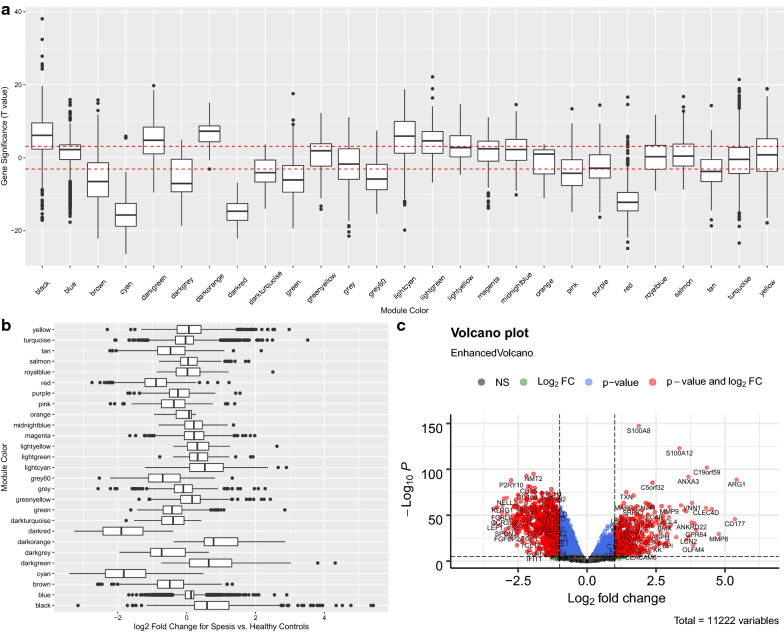
Gene differential expression analysis (sepsis against healthy control) across modules. **a** Boxplot of the distribution of significance *T* values. The black and darkorange modules showed the most significant difference between sepsis versus controls. The red dashed lines indicate statistical significance level of two-tailed T test that have been adjusted by Bonferroni method; **b** fold changes of the differential expression across modules. The darkorange and black modules were among the most up-regulated modules; and **c** volcano plot showing differentially expressed genes between sepsis and healthy controls. Genes such as ARG1, CD177 and MMP8 were most differentially expressed

A soft-thresholding power of 6 was used to obtain approximate scale-free topology for the network (Additional file 1: Figure S3). Consensus WGCNA identified 27 modules (Fig. 1). Gene differential expression analysis between healthy controls and sepsis showed that genes such as ARG1, CD177, MMP8 and C19orf59 were upregulated. Up-or down-regulation of modules were determined by the median of gene significance *T* value and fold changes. For instance, the black model was significantly upregulated with median *T* value > 3 and log2 fold change > 0.5 (Fig. 2).

**Fig. 3:**
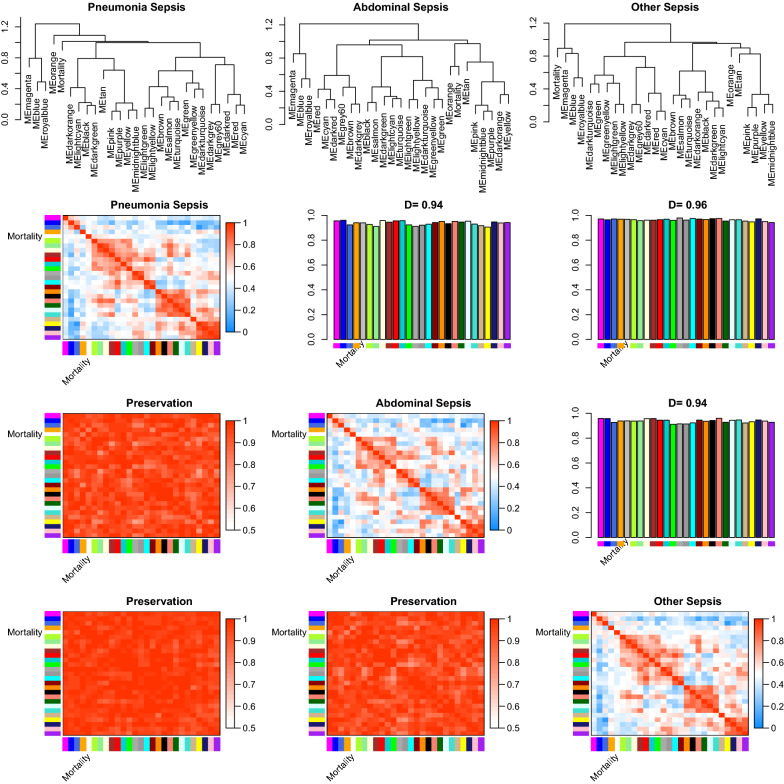
Summary plot of consensus eigengene networks and their differential analysis. The top 3 panels show the dendrograms (clustering trees) of the consensus module eigengenes in the 3 datasets of pneumonia, abdominal and other sepsis. Below, the eigengene networks in the three sets are shown as heatmaps labeled penumonia sepsis, obdominal sepsis and other sepsis. In the heatmaps, red denotes high adjacency (positive correlation) and blue denotes low adjacency. The Preservation heatmap shows the preservation network, defined as one minus the absolute difference of the eigengene networks in the two datasets. The barplot shows the mean preservation of adjacency for each of the eigengenes to all other eigengenes; in other words, the barplot depicts the column means of the preservation heatmap

**Fig. 4:**
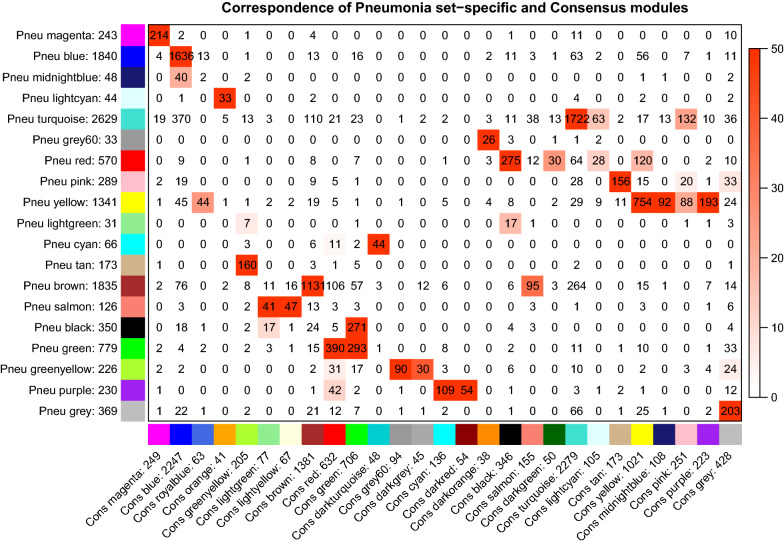
Correspondence of pneumonia set-specific modules and the consensus modules. Each row of the table corresponds to one pneumonia set-specific module (labeled by color as well as text), and each column corresponds to one consensus module. Numbers in the table indicate gene counts in the intersection of the corresponding modules. Coloring of the table encodes − log(p), with *p* being the Fisher’s exact test *p* value for the overlap of the two modules. The stronger the red color, the more significant the overlap is. The table indicates that most peumonia set-specific modules have a consensus counterpart

Consensus eigengene networks and their differential analysis are shown in Fig. 3. The results showed that the eigengene networks in the three datasets were well preserved. To explore whether modules identified in the pneumonia sepsis could also be identified in consensus modules, the correspondence of pneumonia set specific and consensus modules was explored (Fig. 4). The result indicates that most peumonia set-specific modules have a consensus counterpart (Fig. 4). The black color module in the consensus analysis corresponds to the red module in the pneumonia specific set analysis (275 genes overlap with significant *p* value).

### Relating Consensus modules to external clinical traits

**Fig. 5:**
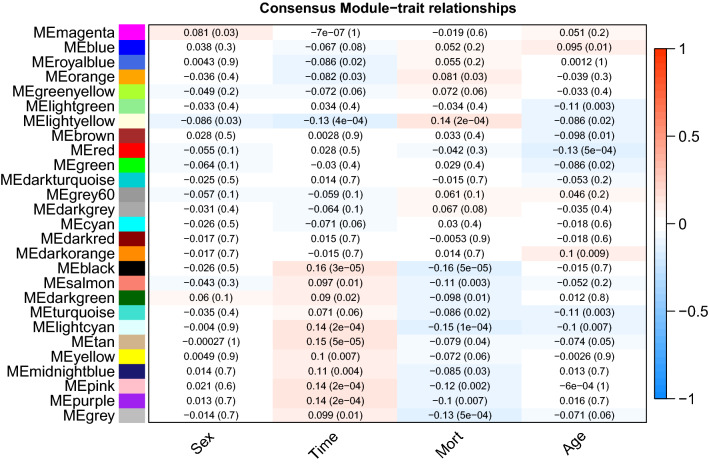
Consensus module-trait relationship. Each module was represented by its eigengene value and correaltion analyses between each of the eigengene values and clinical traits were performed. The first number in the matrix cell represents the corelation coefficient and the number in the parenthesis is the p value for the corelation. The black module was negatively correlated to the mortality and the light-yellow module was positively related to the mortality

The correlation between module eigengene and clinical traits were explored by Pearson’s correlation analysis (Fig. 5). The black module was significantly associated with mortality with higher module express correlated to lower mortality rate (coefficient = -0.16; *p* < 0.001). However, the light-yellow module was positively associated with mortality (coefficient = 0.14; *p* < 0.001). The black module was positively associated with survival time (coefficient = 0.16; *p* < 0.001). The correlations between gene significance for mortality and module membership were explored and the results showed that there was a highly significant correlation between module membership and gene significance for mortality in three modules inclusing the light-yellow, ligh cyan and pink modules (Additional file 1: Figure S4).

### Module biological function

**Fig. 6:**
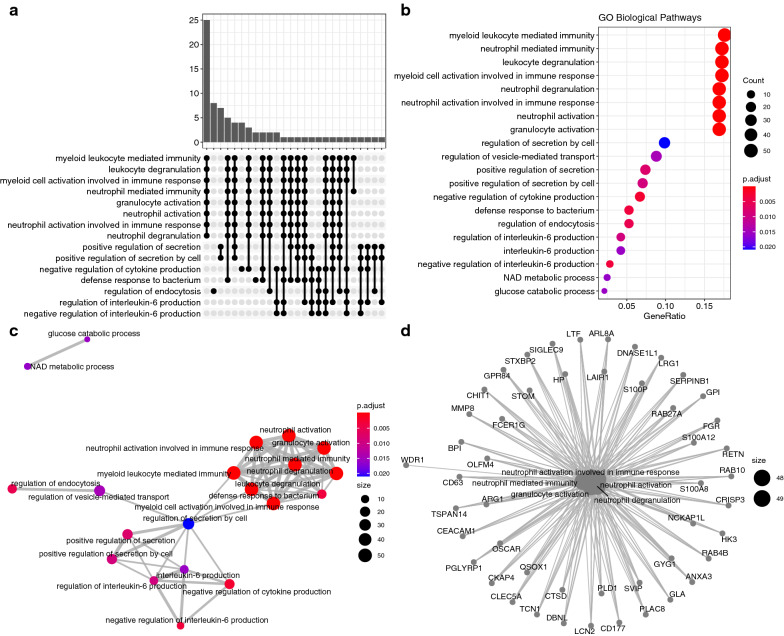
GO enrichment analysis for the black module. **a** The upset plot showing overlap of genes across enriched biological pathways. Biological pathways such as myeloid leukocyte mediated immunity, leukocyte degranulation and neutrophil mediated immunity shared large number of genes. **b** Dot plot showing the ordered enrichment biological pathways; the most enriched pathways included myeloid leukocyte mediated immunity and neutrophil mediated immunity. **c** Enriched terms were organized into a network with edges connecting overlapping gene sets. In this way, mutually overlapping gene sets are tend to cluster together, making it easy to identify functional modules; and **d** The category netplot depicts the linkages of genes and GO terms as a network, which is helpful to see which genes are involved in enriched pathways and genes that may belong to multiple annotation categories. The most important biological pathways in the module involves the immune response, including the immune response including myeloid leukocyte mediated immunity, neutrophil mediated immunity, leukocyte degranulation and myeloid cell activation

**Fig. 7:**
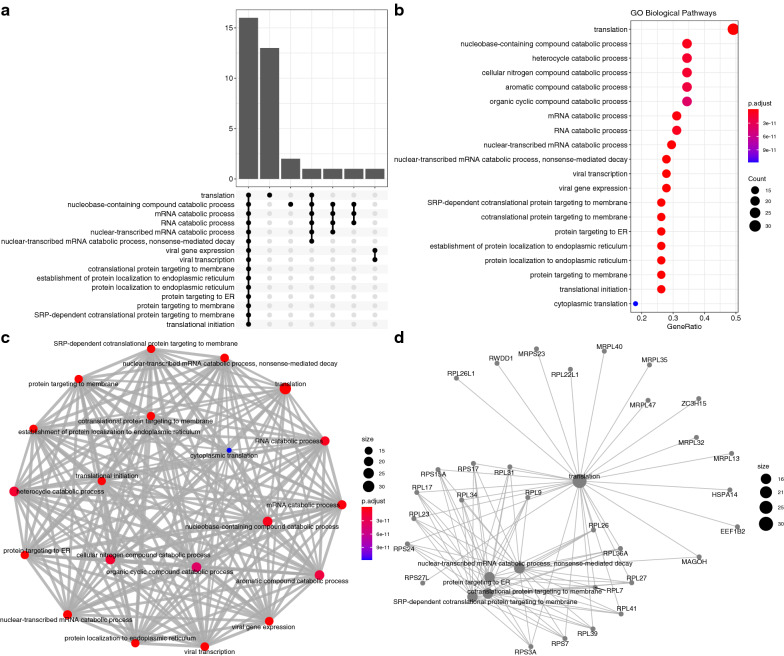
GO enrichment analysis for the light-yellow module. **a** The upset plot showing overlap of genes across enriched biological pathways; **b** dot plot showing the ordered enrichment biological pathways; **c** Enriched terms were organized into a network with edges connecting overlapping gene sets. In this way, mutually overlapping gene sets are tend to cluster together, making it easy to identify functional modules; and **d** The category netplot depicts the linkages of genes and GO terms as a network, which is helpful to see which genes are involved in enriched pathways and genes that may belong to multiple annotation categories. The most important biological pathways in the module involves the translation and catabolic processes, including nucleobase-containing compound catabolic process, heterocycle catabolic process and cellular nitrogen compound catabolic process

The most enriched pathways of the black module included myeloid leukocyte mediated immunity, neutrophil mediated immunity, leukocyte degranulation and myeloid cell activation involved in immune response (Fig. 6). The light-yellow module was enriched in biological pathways such as translation, nucleobase-containing compound catabolic process, heterocycle catabolic process and cellular nitrogen compound catabolic process (Fig. 7).

### Enrichment analysis for transcription factors

Modules were made up of co-expressed genes, indicating that they were regulated by common mechanisms such as the transcription factors. Thus, we performed enrichment analysis for transcription factors. The enrichment analysis involved three steps: (1) motif enrichment analysis with cumulative recovery curves (Additional file 1: Figures S5, S6 and S7), (2) motif-TF annotation, and (3) selection of significant genes. The results showed that the transcription factor CEBPB was the master regulator for the black module, which was annotated to the motif dbcorrdb__CEBPB__ENCSR000BQI_1__m1. A total of 93 genes in the black module was enriched in this motif. The normalized enrichment score (NES) was 5.53. This motif was directly annotated to the transcription factor CEBPB which is an important transcription factor regulating the expression of genes involved in immune and inflammatory responses [25–27]. All enriched motifs and corresponding transcription factors for the black module were shown in Additional file 2: Supplemental Digital Content (SDC) 2. The network of the top 3 enriched motifs and corresponding genes are shown in Additional file 1: Figure S8.

The transcription factor ETV6 was the master regulator for the light-yellow module, which was annotated to the motif taipale__ETV6_full_CCGGAASCGGAAGTN_repr and cisbp__M5425. A total of 12 genes in the light-yellow module were enriched in the motifs. The NES was 6 and 5.98 for these two motifs [Additional file 3: Supplemental Digital Content (SDC) 3].

### Identification of miRNA-target interactions

From the “mirtarbase” table with validated miRNA-target interactions, we identified 1,981 miRNA that were potential regulators of the black module [Additional file 4: Supplemental Digital Content (SDC) 4]. The top 5 miRNA regulated the most number of genes were hsa-miR-335-5p (n = 59), hsa-miR-26b-5p (n = 57), hsa-miR-16-5p (n = 44), hsa-miR-17-5p (n = 42), and hsa-miR-124-3p (n = 38). A network connecting miRNA and target genes are shown in Additional file 1: Figure S9.

For the light-yellow module, we identified 893 miRNA that were potential regulators of the module [Additional file 5: Supplemental Digital Content (SDC) 5]. The top 5 miRNA regulated the most number of genes were hsa-miR-16-5p (n = 14), hsa-miR-92a-3p (n = 12), hsa-miR-26b-5p (n = 9), hsa-miR-615-3p (n = 9), and hsa-let-7b-5p (n = 8).

### Survival analysis

**Fig. 8:**
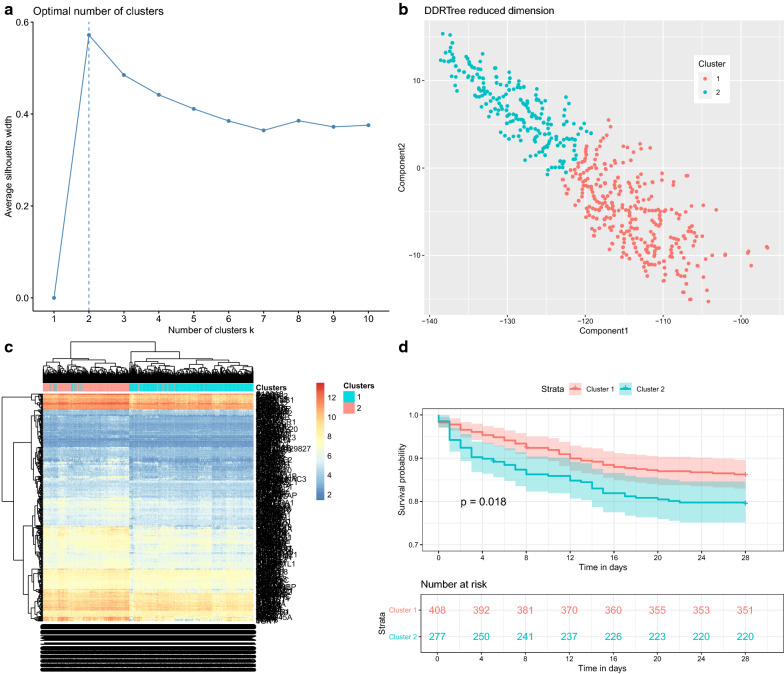
The survival analysis with the black module. **a** Optimal number of classes derived from DDRTree reduced dimension space with the silhouette width; the two-class model was the best fit model with the highest silhouette width. **b** Discriminative dimension reduction (DDR) graph of the black module gene list. K-means clustering was used to separate samples into two groups (blue and red). **c** Heatmap of gene expression sorted by DDR score. The two clusters identified by the DDR score were consistent with that identified by black module genes. Genes showing on the right were those from the black module. **d** Kaplan–Meier curves for groups defined by DDR k-means clustering of black module genes. Cox’s proportional hazard modelling of module groups showed that the cluster membership was significant association with survival (*p* = 0.018)

**Fig. 9:**
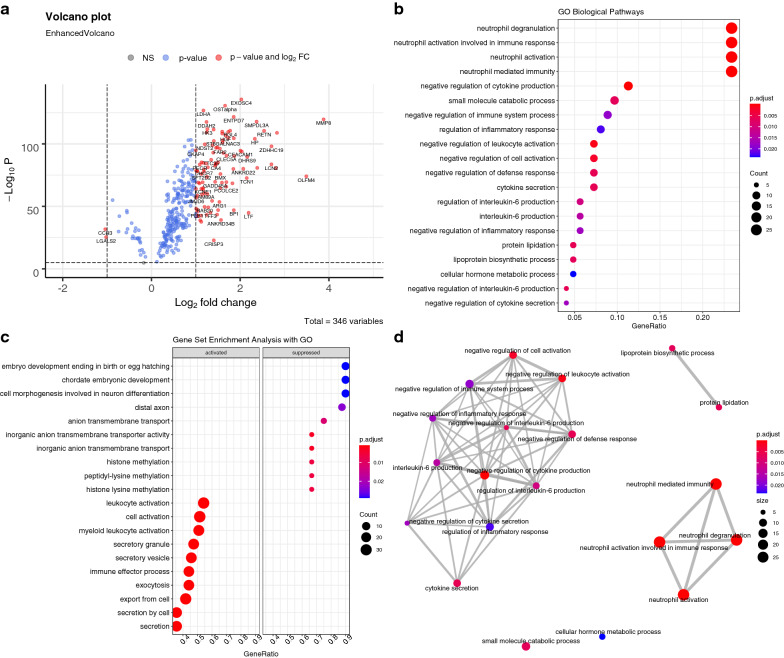
The differences in biological functions between cluster 2 and cluster 1 as identified by the black module. **a** Volcano plot showing differentially expressed genes in the black module between cluster 2 and cluster 1. **b** enriched GO biological pathways. **c** Gene set enrichment analysis with GO term; as compared to cluster 1, cluster 2 was characterized by activated functions such as leukocyte activation, cell activation and myeloid leukocyte activation. **d** Enrichment Map for enrichment result of over-representation test

To further validate that the black module was related to the mortality outcome, the high-dimension space was reduced to lower dimension space by using reversed graph embedding (DDRTree). The method provided non-linear dimension reduction that retains the intrinsic structure of the sample data. The silhouette method showed that 2-cluster would be the optimal number (Fig. 8a). In the 2-dimension space (Fig. 8b), the samples were classified into two clusters. Heatmap of all module genes showed that the two clusters could be well separated (Fig. 8c), which supported that the intrinsic structure was well preserved with the dimension reduction. The two clusters were significantly associated with survival outcome (p = 0.018 in the Cox proportional hazard model, Fig. 8d). Cluster 2, as compared with Cluster 1, was characrized by activated functions including myeloid cell activation, cell activation, leukocyte activation and myeloid leukocyte activation (Fig. 9).

**Fig. 10:**
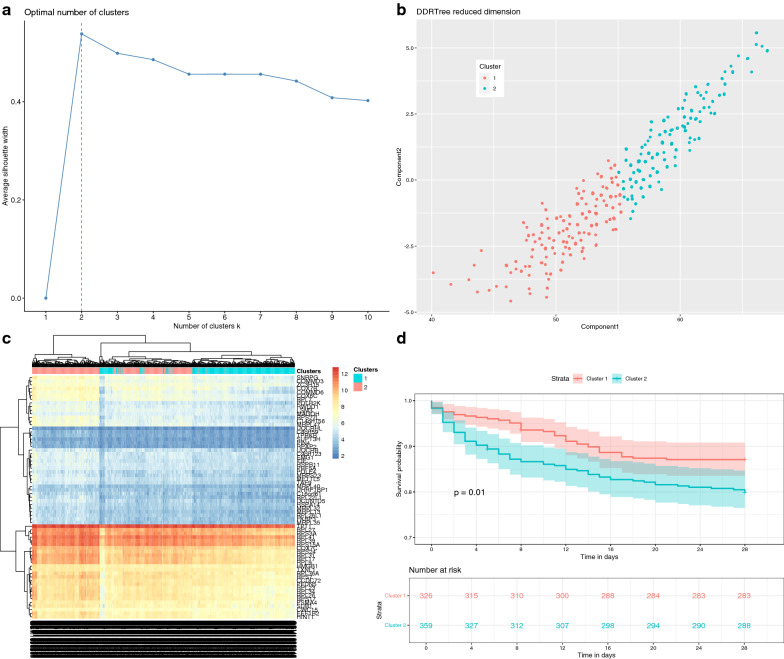
The survival analysis with the light-yellow module. **a** Optimal number of classes derived from DDRTree reduced dimension space with the silhouette width; the two-class model was the best fit model with the highest silhouette width. **b** Discriminative dimension reduction (DDR) graph of the light-yellow module gene list. K-means clustering was used to separate samples into two groups (blue and red). **c** Heatmap of gene expression sorted by DDR score. The two clusters identified by the DDR score were consistent with that identified by light-yellow module genes. Genes showing on the right were those from the light-yellow module. **d** Kaplan–Meier curves for groups defined by DDR k-means clustering of light-yellow module genes. Cox’s proportional hazard modelling of module groups showed that the cluster membership was significant association with survival (*p* = 0.01)

The light-yellow module was also related to the mortality outcome. The silhouette method showed that 2-cluster would be the optimal number (Fig. 10a). The study population could be classified into two clusters in a 2-dimension space. Heatmap (Fig. 10b, c) showed that the study population could be well separated into two clusters. Cluster 2 showed significantly lower survival probability than cluster 1 (*p* = 0.01, Fig. 10d).

## Discussion

The study employed consensus network analysis to identify gene co-expression modules. These modules were involved in distinct biological functions and were associated with clinical traits. Regulatory mechanisms of some important modules involving transcription regulators and miRNA were explored by validated databases. Consistent with previously published studies employing high-throughput dataset to examine sepsis, these modules were enriched for pathways related to immune response [28, 29]. The black module was significantly associated with survival outcome and the transcription factor CEBPB was the master regulator of this module. Several miRNAs including hsa-miR-335-5p, hsa-miR-26b-5p, hsa-miR-16-5p, hsa-miR-17-5p and hsa-miR-124-3p were identified to be important regulators of the gene expressions in the module.

Our analysis has several implications for research and clinical practice. First, sepsis is shown to have a dysregulated immune response highlighted by the upregulation of the black module involving biological functions such as myeloid leukocyte mediated immunity, neutrophil mediated immunity, leukocyte degranulation and myeloid cell activation involved in immune response. Immune dysfunction has long been recognized as an important mediator of sepsis [4, 30, 31]. However, previous studies mostly analyzed high-throughput data at individual gene level involving differential expression analysis followed by functional pathway enrichment [4, 15, 31, 32]. In neonate sepsis, Meng and colleagues identified 7 hub genes in key pathways. However, the study did not relate these gene expression profiling to clinical outcomes and the results cannot be extrapolated to adult sepsis [33]. The present study employed WGCNA to identify modules associated with mortality by treating co-expressed genes as a module. The idea is that genes are working together to take their functions in disease processes. Second, by focusing on modules most significantly associated with mortality, we identified several important regulators including transcription factors and miRNAs. The results support the hypothesis that co-expressed genes are very likely to be regulated by common factors. These transcription factors (such as CEBPB and ETV6) and miRNAs are potential targets for the treatment of sepsis. Since the upregulation of the black module is significantly associated with mortality, drugs targeting these sites may improve the survival outcome. Third, although sepsis is a heterogeneous syndrome [34–38], different types of sepsis share some common important immune regulatory pathways. Our study employed consensus WGCNA and examined whether the module identified in one cause of sepsis can also be found in other causes of sepsis. The results show that most modules are well preserved despite the various causes of sepsis, indicating common pathways associated with the sepsis. Furthermore, the module network constructed by TOM is also well preserved across various causes of sepsis. Collectively, these findings support the notion that sepsis can be considered as a clinical symdrome because various causes of sepsis lead to common pathways. Drugs designed to target these common pathways can be helpful in improving clinical outcomes. For example, our study showed that cluster 2 identified by the black module was associated with lower survival probability, and this subtypes of sepsis was characterized by leukocyte activation. Such over-activation of inflammatory response may indicate that immunoregulatory agents such as arachidonic acid and eicosapentaenoic acid can help to improve the survival outcome [39].

Mortality is an important clinical trait and thus we tried to identify modules most significantly assocated with mortlaity. Two methods were employed to validate that the black module was associated with mortality. Firstly, we simply correlate the module eigengene to the mortality. Module eigengene is computed as the first component in principal component analysis (PCA), which however is a linear transformation of the high-dimensional space. It is well recognized that linear transformation cannnot fully recover the intrinsic structure of a high-dimensional space [40–42]. Thus, we also emplyed manifold learning to better capture the black module [24]. The result is consistent with the above observation that the black module can well separate survivors from non-survivors. Most clinical trials of sepsis are focusing on how to reduce mortality. The identified black module in our study can help to design drugs that potentially useful for reducing mortality rate. For example, our analysis shows that hsa-miR-335-5p is the top ranked miRNA in the regulation of the black module. Since the black module consists mostly genes involved in inflammatory response, the upregulation of hsa-miR-335-5p is proposed to have inflammatory suppression effects [43–45]. More recently, hsa-miR-335-5p is shown to reduce inflammation via negative regulation of the TPX2-mediated AKT/GSK3β signaling pathway in a chronic rhinosinusitis mouse model [46]. Collectively, these observations strongly support our results that hsa-miR-335-5p can be a candidate therapeutic target.

The CEBPB was annotated to the most significantly enriched motifs for genes in the black module. The eigengene of black module is the most significant associated with mortality, and thus the master regulator CEBPB is also an important risk factor for mortality via immunomodulation [47, 48]. Our finding is also consistent with a recent study comparing sepsis with and without shock, in which CEBPB is significantly enriched in septic shock versus non-shock patients [49]. Since the presence of shock is a significant risk factor for mortality, the result supports the notion that CEBPB can be a potential target for improving survival outcome.

The strength of the study is the large sample size. To the best of our knowledge, the MARS consortium is the largest sepsis cohort with genome-wide blood transcriptional profiling [16]. Furthermore, the included sepsis subjects were classified into different causes of sepsis, allowing for the consensus network analysis. The consensus analysis identified common functional modules involved in sepsis. However, there are limitations in the study. First, the severity of illness is not available in the cohort, prohibiting correlation analysis for modules and severity scores. However, since most severity scores are developed with the mortality as the end point, the mortality can be used as a surrogate for the severity of illness. Second, the transcription regulators were predicted by bioinformatic analysis, which might have high false positive rate. The in vivo function of these transcription factors and miRNAs should be validated in experimental studies. In this regard, results from the current analysis can be considered as hypothesis-generating. Third, there are more causes of sepsis that have not been categorized in the study. For example, urinary tract infection is also an important cause of sepsis in clinical practice, however, the dataset did not contain this subclass of sepsis.

## Conclusion

In conclusion, the present study identified the black and light-yellow modules to be the most significantly associated with mortality. Master regulators of the black module included transcription factor CEBPB. miRNA-target interactions identified significantly enriched miRNA. These regulators can be potential therapeutic targets for the treatment of sepsis.

## Supplementary information

**Additional file 1.** Supplemental digital content 1.

**Additional file 2. **Supplemental digital content 2.

**Additional file 3.** Supplemental digital content 3.

**Additional file 4.** Supplemental digital content 4.

**Additional file 5.** Supplemental digital content 5.

## Data Availability

Data were available on request.

## Abbreviations

GEO: Gene Expression Omnibus
WGCNA: Consensus weighted gene co-expression netwoek analysis
ICU: Intensive care unit
TOM: Topological overlap matrices
GO: Gene Ontology
TF: Transcription factor
TSS: Transcription start site
