# Supplementary material for: Gene correlation network analysis to identify regulatory factors in sepsis
Source: J Transl Med. 2020 Oct 8;18:381. doi: 10.1186/s12967-020-02561-z (PMC7545567; doi:10.1186/s12967-020-02561-z)
Supplement: Supplementary file 1 — Additional file 1. Supplemental digital content 1. [file 12967_2020_2561_MOESM1_ESM.docx]

Supplemental Digital Content

Figure S1. MA plot of sample arrays.

M and A are defined as: M = log_2_(I_1_) - log_2_(I_2_)

A = 1/2 (log_2_(I_1_)+log_2_(I_2_)),
where I_1_ is the intensity of the array studied, and I_2_ is the intensity of a "pseudo"-array that consists of the median across arrays. Typically, we expect the mass of the distribution in an MA plot to be concentrated along the M = 0 axis, and there should be no trend in M as a function of A. If there is a trend in the lower range of A, this often indicates that the arrays have different background intensities; this may be addressed by background correction. A trend in the upper range of A can indicate saturation of the measurements; in mild cases, this may be addressed by non-linear normalisation (e.g. quantile normalisation).

Figure S2. Sample clustering on all genes statified by the causes of sepsis. Outliers were excluded from subsequent analysis.

Figure S3. Summary network indices (y-axes) as functions of the soft thresholding power (x-axes). Numbers in the plots indicate the corresponding soft thresholding powers. The plots indicate that approximate scale-free topology is attained around the soft-thresholding power of 6 for the three sets. Because the summary connectivity measures (mean, max and median) decline steeply with increasing soft-thresholding power, it is advantageous to choose the lowest power that satisfies the approximate scale-free topology criterion (R2 > 0.8).

Figure S4. Scatterplots of gene module membership and gene significance for mortality. Each point in the plots represent a gene in the relevant module. Gene significance for mortality was tested by comparing gene expression levels between survivors and non-survivors. Module membersip was calculated by correlating gene expression profile with the module eigengene of a given module. There is a highly significant correlation between module membership and gene significance for mortality in three modules inclusing the light yellow, ligh cyan and pink modules. Unfortunately, the significance was not statistically significant in the black module.

Figure S5. Histogram of the Area under the cumulative recovery curve. The first step to estimate the over-representation of each motif on the black module gene-set is to calculate the Area Under the Curve (AUC) for each pair of motif-geneSet. This is calculated based on the recovery curve of the gene-set on the motif ranking (genes ranked decreasingly by the score of motif in its proximity, as provided in the motifRanking database). The red vertical line indicates the significance level that motifs with a AUC greater than the significance level are considered significant motifs.

Figure S6. Histogram of the Area under the cumulative recovery curve. The first step to estimate the over-representation of each motif on the light-yellow module gene-set is to calculate the Area Under the Curve (AUC) for each pair of motif-geneSet. This is calculated based on the recovery curve of the gene-set on the motif ranking (genes ranked decreasingly by the score of motif in its proximity, as provided in the motifRanking database). The red vertical line indicates the significance level that motifs with a AUC greater than the significance level are considered significant motifs.

Figure S7. Cumulative recovery curve for a few motifs. The red line represents the global mean of the number of recovered genes and the green line represents the 3 SD. Motifs greater than the 3SD were considered statistically significant. Thus, the motifs dbcorrdb__CEBPB__ENCSR000BQI_1__m1 and cisbp__M4591 were significantly enriched for the black module. The transfac_pro__M00770 motif did not reach the significance level.

Figure S8. Top 3 enriched motifs in the black module. The large blue circles indicate the motifs and small red circles indicate the candidate genes.

Figure S9. Top 5 miRNA and their target genes identified from the “mirtarbase” table.
